# A Scientometric Analysis of Global Health Research

**DOI:** 10.3390/ijerph17082963

**Published:** 2020-04-24

**Authors:** Minxi Wang, Ping Liu, Rui Zhang, Zhi Li, Xin Li

**Affiliations:** College of Management Science, Chengdu University of Technology, Chengdu 610059, China; wangminxi@mail.cdut.edu.cn (M.W.); liupingcdu@163.com (P.L.); ruizhang033@163.com (R.Z.); leannlee0618@gmail.com (Z.L.)

**Keywords:** global health, public health, scientometric study, knowledge map, visualization analysis, CiteSpace

## Abstract

With the development and deepening of the process of global integration, global health is gaining increasing attention. An increasing number of studies have examined global health from diverse perspectives to promote the realization of global public health. The purpose of this research is to systematically and comprehensively evaluate the knowledge structure, knowledge domain, and evolution trend in the field of global health research. Based on the 14,692 document data retrieved from Web of Science Core Collection from 1996 to 2019, this article carried out a visual analysis of global health research from the perspective of scientific output characteristics, scientific research cooperation networks, keywords, and highly cited literature. The results show that scholars’ interest in global health research is increasing, especially after the outbreak of SARS. USA, England, Canada, Australia, and China have the most prominent contributions to global health research. Significant authors, high impact journals and core institutions also identified. The study found that “global health governance”, “global health diplomacy”, “medical education”, “global health education” and “antimicrobial resistance” are the research frontiers and hot spots. This study provides an overview and valuable guidance for researchers and related personnel to find the research direction and practice of global health.

## 1. Introduction

Globalization has accelerated the spread of health risks, and the health threats of a certain country or region may become a global problem in a short time. Therefore, the health status of the public in one country is not only determined by the political, economic, and cultural development of the land but also affected by the health and safety status of other countries in the world [1]. Due to the globalization, complexity, and diversification of health influence factors, the development of global health requires all-round cooperation from all countries in the world. Global health, in a broad context, refers to improving public health worldwide, reducing disparities, and protecting against global threats that do not consider national borders [2].

Since the 21st century, infectious diseases, chronic diseases, climate change, resource depletion, ethnic conflicts, and poverty have continuously threatened the health of the global public [3]. Since the outbreak of the new coronavirus in 2019 (COVID-19), the number of people infected with the virus worldwide has reached more than 2.1 million, causing more than 145,000 deaths, and the epidemic has spread to 211 countries (as of 15 April 2020) [4]. The new coronavirus epidemic has brought tremendous harm and threat to the health of the global public and also exposed many problems in global health governance [5]. In this context, the global experts and scholars’ attention to global health likely will further increase. To promote the deepening of research in global health, it is particularly essential to comprehensively summarize and review the current research results in the field of global health. Gostin et al. introduced the development history, framework, and deficiencies of global health law, and sought to establish domestic and global links in the field of health law [6]. Dieleman et al. examined the status and characteristics of fiscal health expenditures in 195 countries/regions and predicted the future development trend of global health spending [7]. García et al. revealed the severity and causes of corruption in the global health system, noting that policymakers, research scholars, and funders need to clarify their responsibilities and treat corruption as an essential area of research [8]. Herath et al. combed the research literature of interprofessional education in global health care and analyzed the main differences and features of global health education for undergraduates and postgraduates in developed and developing countries [9]. Other scholars have reviewed different aspects of global health, such as experience and progress in the prevention and control of infectious diseases [10], lessons learned and theoretical basis for the implementation of global health security [11], and mechanisms for international institutions to participate in global health governance [12]. Most of the existing studies started from a single perspective or focused on a specific research area of global health, which lacks a comprehensive and systematic review of global health research.

Science is not an independent activity. Therefore, the progress of science often needs to summarize previous research results [13]. In the past, the number of research literature on global health has multiplied [14,15]. Because global health research involves multiple disciplines and scattered research themes, it has brought a lot of difficulties for global health researchers to grasp this emerging research area and find research hotspots. Due to the development of technologies such as data mining, information analysis, and graphic drawing, the organic combination of computer technology and traditional mathematical statistics has made it possible for visual analysis of scientific metrology. Scientometrics can intuitively show the information panorama of each discipline through the knowledge map, and explore research hotspots and emerging trends in a particular field [16]. It is widely used in the areas of environmental ecology [17], public health [18,19], business economics, artificial intelligence [20], education research, resource science [21], and medicine.

From a new perspective, this study will use scientometric to comprehensively and systematically review the research in the field of global health. Specific analysis methods such as text mining, word frequency analysis, co-word analysis, cluster analysis, co-citation analysis, and network analysis will be adopted in this research to answer the following questions: (1) What are the changes in international experts’ and scholars’ attention to global health? (2) Which scholars, research institutions, countries, or regions have outstanding influence and contribution to the development of global health research? (3) Which journals have a high impact in the area of global health research? (4) What is the status of scientific research cooperation across global, multi-institution, and different authors in global health? (5) What is the evolutionary context, research frontiers, hot topics, and future trends in global health research?

## 2. Materials and Methods 

### 2.1. Data Source 

**Figure 1:**
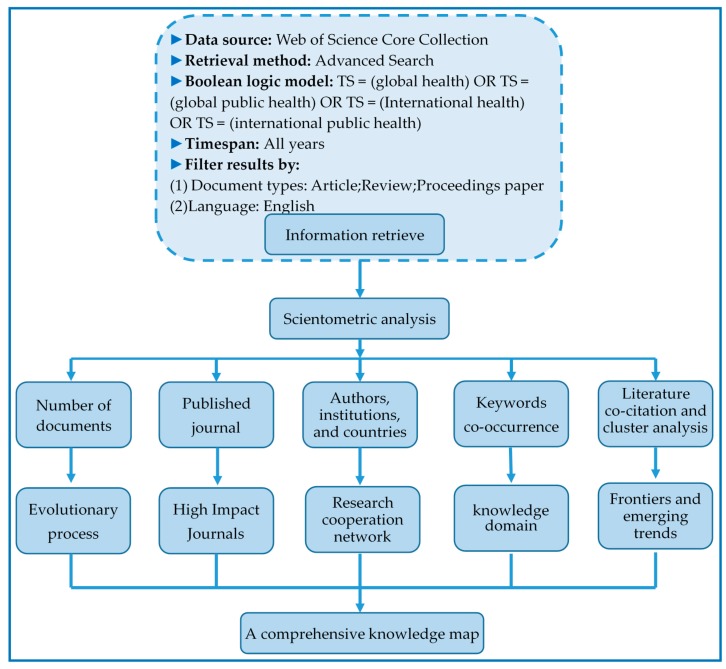
Research framework.

Literature databases commonly used by international experts and scholars include Google Scholar, Scopus, PubMed, and Web of Science. Each of these databases has advantages and disadvantages. Google Scholar has a broad coverage of literature data and plentiful literature types, but it has low data quality and many duplicate data [22]. PubMed is a free biomedical information retrieval system developed by the NCBI. PubMed has a rich literature in the medical field but lacks literature data in other subject areas. Scopus and Web of Science are comprehensive databases, and there is not much difference in data coverage between the two. However, some scholars have shown that when using CiteSpace software for visual analysis, the knowledge map made by the literature of the Web of Science database is better [23,24]. Therefore, this study uses the Web of Science to retrieve the literature data needed for analysis. To ensure the reliability of the scientific metrological analysis, we chose the Web of Science core collection, and the indexes are SCI-EXPANDED, SSCI, CCR-EXPANDED, and Index Chemicus. The detailed retrieval strategy is shown in Figure 1. After preliminary searching, 14,692 pieces of literature data were obtained. For some reason, there are differences and ambiguities in the expressions of author names, institution names, and country names in literature data. In response to this, before conducting scientific measurement analysis, we used CiteSpace’s data deduplication and name merge functions to standardize the data. The literature search date is December 30 December 2019.

### 2.2. Data Visualization and Analysis

The software used in this article for scientometric analysis and visualization analysis is CiteSpace (5.6.R2). CiteSpace software is an information visualization software developed by Chen Chaomei, based on the Java language [25]. CiteSpace’s theoretical basic system mainly includes five aspects: Kuhn’s scientific development model theory, Price’s scientific frontier theory, structural tree holes, the best information foraging theory of scientific communication, and the theory of discrete and reorganized knowledge units [26]. This article uses CiteSpace software to visualize the structure, regularity, and distribution of knowledge in the global health field, and analyze the co-citation of documents to mine the knowledge clustering and distribution of citation space. At the same time, we also performed co-occurrence analysis between other knowledge units in the global health field, such as cooperation between authors, institutions, and countries. Finally, we built a comprehensive knowledge map of global health research based on the results of scientific econometric analysis. Figure 1 shows the research framework of the article.

Some parameters and knowledge map identification methods will be involved in the results of the scientometric analysis, which will be explained uniformly here. The knowledge map shows the distance of time with warm and cold colors. When the time is closer to 2019, the colors become warmer. In the knowledge graph, the size of the nodes means the frequency of the authors, institutions, countries, and journals, and the connection between the nodes indicates that these nodes appear in the same article [27]. In general, when two or more authors (institutions, countries) appear in the same paper, it can be regarded as a scientific research cooperation relationship between these authors (institutions, countries) [28]. In the process of scientometric analysis, there are also some parameter indicators for a specific evaluation. H-index is a mixed quantitative index proposed by physicist George Hirsch of the University of California, USA, which is used to evaluate the amount of academic output and the level of the scholarly output of researchers and institutions. H-index indicates that h of the N papers published in the journal have been cited at least h times [29]. The Degree in the table indicates the number of connections between authors (institutions, countries) in the co-occurrence knowledge graph. A higher Degree value indicates more communication and cooperation between the authors (institutions, countries). Besides, intermediary centrality is an indicator that measures the importance of nodes in the research cooperation network, and the half-life is a parameter that represents the continuity of institutional research from a time perspective [25].

## 3. Results

### 3.1. Progression of Scientific Output

**Figure 2:**
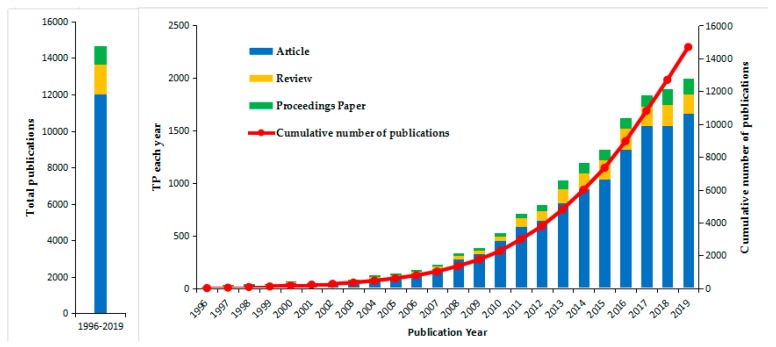
The scientific output from 1996–2019. The number of publications each year is based on the main ordinate axis (the left ordinate), and the cumulative number of publications is based on the secondary axis (right ordinate).

The change in the number of scientific research results reflects, to a certain extent, the changes in the attention paid by international experts and scholars to a specific subject area. A total of 14,692 literature data on global health research were recovered, including 12,012 articles, 1627 reviews, and 1053 proceedings papers. Figure 2 shows the details of the scientific output. The number of publications rose from 14 in 1996 to 1997 in 2019. On the whole, the scientific production in global health research shows a continuous upward trend. Specifically, the three types of documents (article, review, and proceedings paper) are also showing a growing trend.

### 3.2. Analysis of Journals

**Figure 3:**
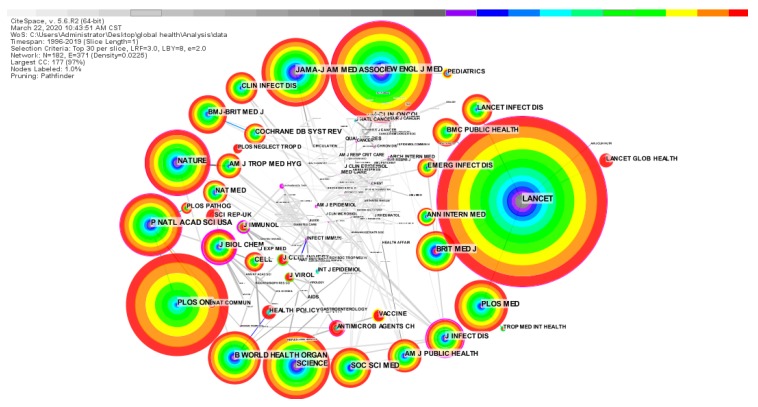
Visualization of co-citation journals.

Compared with the number of publications, the frequency of citations to the literature published by a journal can better reflect the influence and importance of the journal. Therefore, this article uses CiteSpace to analyze the quote of the journals, and produced a map of the cited journals, as shown in Figure 3. Based on the frequency of citations, we selectively counted the detailed information of the top 20 journals and drew Table 1. As can be seen in Figure 3 and Table 1, after careful consideration of citation frequency, impact factor, centrality, and H-index, the top five core journals are LANCET (IF: 59.10, H-index: 700), NEW ENGL J MED (IF: 70.67, H-index: 933), JAMA-J AM MED ASSOC (IF: 51.27, H-index: 622), SCIENCE (IF: 41.03, H-index: 1058) and NATURE (IF: 43.07, H-index: 1096). In Figure 3, the nodes of LANCET, NEW ENGL J MED, JAMA-J AM MED ASSOC, SCIENCE, NATURE, and P NATL ACAD SCI USA have relatively large node circles, and there are cool-tone areas in the node circles. However, node circles such as PLOS ONE, BMC PUBLIC HEALTH, LANCET INFECT DIS, and LANCET GLOBAL HEALTH are mostly warm colors. It shows that the critical early literature on global health research mainly came from the journals of LANCET, NEW ENGL J MED, JAMA-J AM MED ASSOC, SCIENCE, NATURE, and P NATL ACAD SCI USA. It is worth noting that the top five journals in the global health field are from the United States (NEW ENGL J MED, JAMA-J AM MED ASSOC, SCIENCE ) and the United Kingdom(LANCET, NATURE).

### 3.3. Analysis of Scientific Cooperation Network

#### 3.3.1. Co-author Analysis

**Figure 4:**
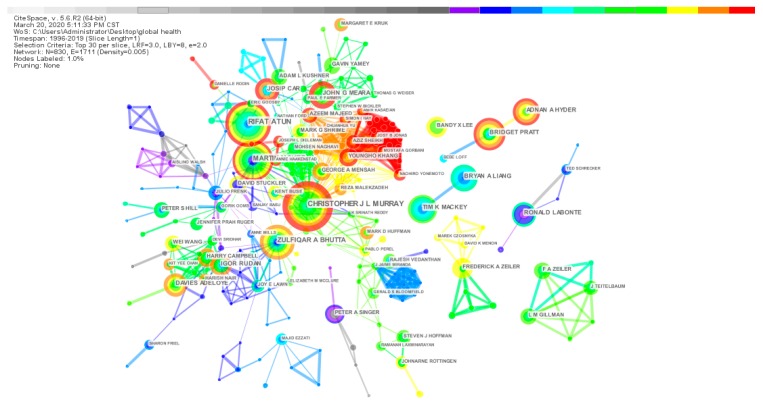
Knowledge map of co-author collaboration network.

As shown in Figure 4, the author’s co-occurrence network knowledge graph has 830 nodes and 1171 connections. On the whole, the author’s cooperation network density value is only 0.005, and the overall global cooperation and communication still need to be strengthened.

The scientific research cooperation group centered on the authors of MURRAY C J L, ATUN R, MCKEE M, BHUTTA ZA, and MEARA JG has the closest communication. The details of the top 20 authors published papers are shown in Table 2.

In Table 2, MURRAY C.J.L. is the scholar with the most significant number of published papers in global health research, with a degree value of 49 and an H-index of 120. MURRAY C.J.L. mainly focuses on global health burden research, global health international assistance, global health finance, infectious disease prevention, global health professional education gap, etc. [30,31,32]. The research findings of ATUN R. are mainly related to the interaction between global health initiatives and national health systems, cancer control in low-income countries, measures to improve health systems in developing countries, and innovations in health financing [33,34,35]. The dialectical relationship between economic development and global health, global health diplomacy, and the strengthening of the global health system are the main concerns of MCKEE M. [36,37]. The two remaining core authors in the top five are BHUTTA Z.A. and PRATT B.

#### 3.3.2. Co-institution Analysis

**Figure 5:**
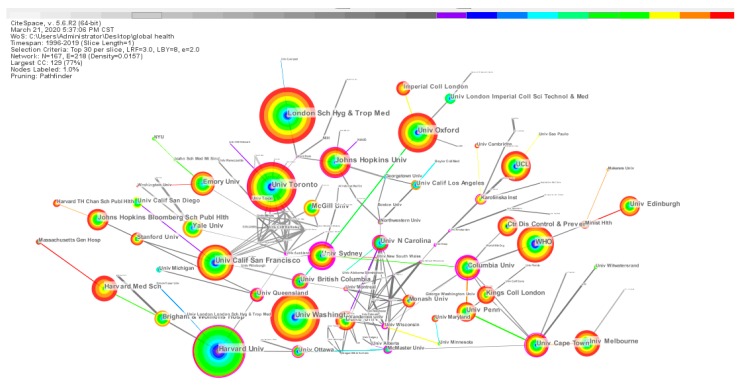
Knowledge map of co-institution collaboration network.

Figure 5 presents a co-institution network consisting of 167 nodes and 218 links. It can be seen from Figure 5 that there is a certain amount of collaboration and exchange between institutions in the world, but the cooperation between domestic institutions is closer than that between international institutions.

Combined with Figure 5, the top 20 institutions with published papers are listed in Table 3. The London Sch Hyg and Trop Med (433 articles, Harvard Univ (409 articles), Univ Toronto (396 articles), Univ Washington (385 articles), and Univ Oxford (327 articles). All of them made central contributions to the research on global health. It is worth noting that 12 of the top 20 institutions are from the USA, with 11 schools and one government agency. The second is the UK, with three institutions (London Sch Hyg and Trop Med, Univ Oxford, and UCL) in the top 20. It further demonstrates the outstanding contributions and leadership role of the USA and the UK in the field of global health research.

#### 3.3.3. Co-country/Territory Analysis

**Figure 6:**
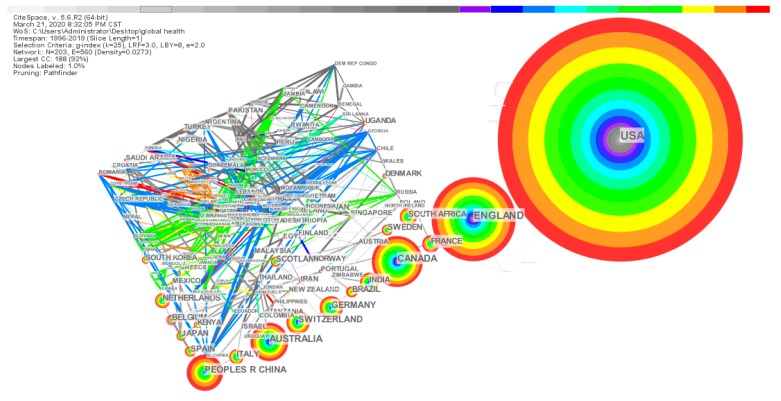
Knowledge map of the co-country/territory collaboration network.

In Figure 6, the country/territory cooperative network map has 203 nodes, 560 links, and the network density is 0.0273.

From a global perspective, the network density of knowledge graphs is high, there are many connections between countries, and cooperation and exchanges between countries are relatively close. It also shows that global health has attracted the attention of various countries around the world. Due to the differences in the political, economic, and cultural development of each country, the coordination of global health and domestic health systems is that these countries also tend to solve problems in coordination and dialogue with other countries. In Table 4, it can be seen that the USA is the country with the most significant number of papers published in the global health field, with 6561 articles published accounting for 44.7% of the total.

The remaining top five are the ENGLAND (2635 articles, 16.1%), CANADA (1504 articles, 10.2%), AUSTRALIA (1108 articles, 7.5%), and PEOPLES R CHINA (1086 articles, 7.3%). From Figure 6, the center of the circle of this node in PEOPLES R CHINA is mainly a warm tone, while the center of the circle of the node of the USA and ENGLAND has a cold tone. It shows that China, as a developing country, started research in the global health field later than developed countries such as the United States. Besides, although there are already 1086 articles published in China, among the top 20 authors and institutions, there are no authors and institutions from China. It indicates that although China started late in the global health field, it has developed rapidly.

### 3.4. Analysis of Co-occurring Keywords

After the vital co-occurrence network analysis, the keywords are summarized and classified according to the keyword frequency and research direction, as shown in Table 5.

These manually selected keywords are roughly divided into five main topics. The keywords of topic one mainly include “global health (2583)”, “quality of life (933)”,” mortality (712)”, “public health (535)”, “survival (200)”, “health status (52)”. Topic 1 is the main goal and direction of global health research, namely reducing global abnormal mortality through global health governance and international cooperation, meeting the minimum survival needs of people around the world, gradually enhancing the health status, and continuously improving the quality of life of the public. The second major topic in global health research is the threat factors that specifically affect global public health, including “infectious diseases (779)”, “cancer (355)”, “antibiotic resistance (39)”, “mental health issues (50)”, “obesity (348)”, “climate change (56)”, etc. The health prevention and treatment of some specific groups (children, women, students) is the third topic focused by scholars in the global health field. The keywords included in the fourth topic are “developing countries (399)”, “Africa (384)”, “middle-income country (62)”, and the United States (348). The fifth is a relatively macro topic, mainly on the cooperation mechanism of global health, global health management, prevention of global health risks, and professional education of global health.

### 3.5. Literature Co-citation Analysis

#### 3.5.1. Analysis of Highly Cited Literature

**Figure 7:**
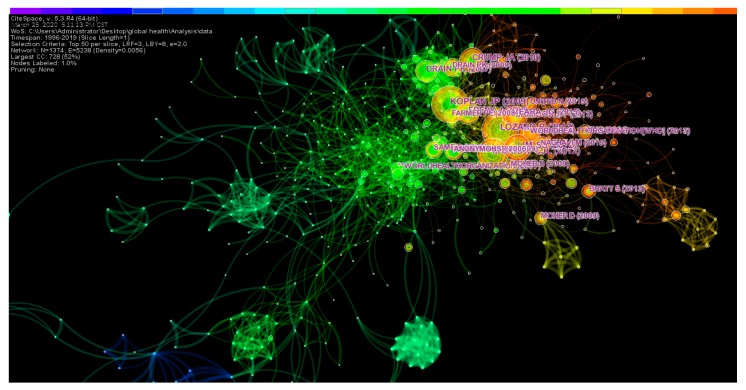
Knowledge map of co-citation literature. The selection criteria for the study of literature co-citation networks is top 50 per slice, and the largest citation sub-network is displayed.

Usually, scholars cite the research results of their predecessors in their papers and list them in the form of references. Mutual citations of scientific literature reflect the objective laws of scientific development [38]. In Figure 7, the early citation network is relatively sparse, and the middle and late citation networks are denser. At the same time, it can be seen from the location of some large nodes that highly cited documents also appear in the middle and late periods.

With reference to Figure 7, this study selected the top ten documents cited by frequency and detailed information of these documents listed in Table 6. It should note that the citation frequency in this article is limited to the mutual citation between these 14,692 articles so that the specific citation frequency will be different from the statistics in Web of Science. The article “Towards a common definition of global health” published by Koplan J.P. is cited most frequently [2]. About the term “global health”, the academic community has not unified its final definition. At present, the explanation given by Koplan J.P. scholars recognized by the academic community. The burst value in the last column of the table indicates that the literature has received significant attention for a certain time. The highest burst value is the article “Global and regional mortality from 235 causes of death for 20 age groups in 1990 and 2010” published by Lozano R. et al. [39]. The rest of the literature is mainly on global health disease burden research, global health status survey, medical education, and global surgical exploration. Eight of the top ten most frequently cited articles are from the Lancet journal.

#### 3.5.2. Cluster Analysis of Literature Co-citation Network

**Figure 8:**
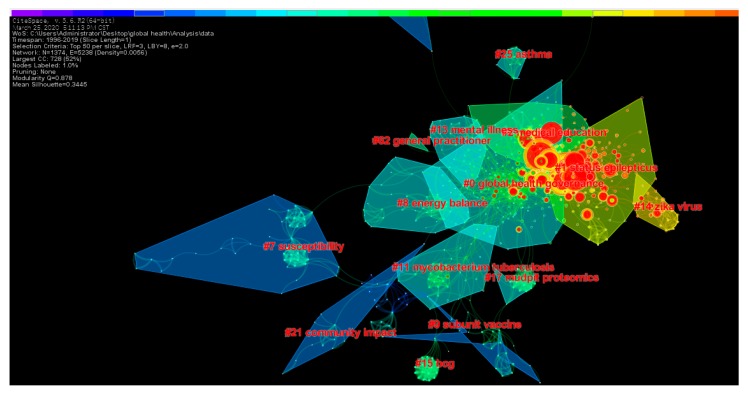
Knowledge map of literature cluster. In CiteSpace, clusters are named by citing the keywords of the literature, and the log-likelihood algorithm (LLR) used.

As an exploratory data mining technology, cluster analysis used to analyze and determine important topics, content, and evolution trends. Cluster analysis of literature co-citation can effectively classify a large number of similar research documents into a single knowledge unit, and then objectively reflect the main content of each knowledge unit [47]. The literature clustering knowledge map is shown in Figure 8.

In Figure 8, the color of the cluster blocks from cold to warm represents the average time of clustering from far to near. The red nodes in the cluster color block represent the literature with burst value. The more red nodes in the cluster block, it shows that this clustering topic is the research frontier and hot spot. In order to further understand the clustering theme, we have summarized the detailed information of clustering and plotted it as Table 7.

It can be seen from Table 7 that the silhouette values of all clustering results are greater than 0.7, indicating that there is no problem with clustering. According to Figure 8 and Table 6, currently, “global health governance”, “global health diplomacy”, “medical education”, “global health education”, “antimicrobial resistance” and “Zika Virus” are the research frontiers and hot spots in the global health field. In addition, “quality of life”, “energy balance; adipose tissue; appetite”, “subunit vaccine”, “mental illness”, “mental health”, “community impact”, “health services” and “interaction strategies” are important research topics and directions of global health.

### 3.6. Category Co-occurrence Analysis and a Knowledge Map

**Figure 9:**
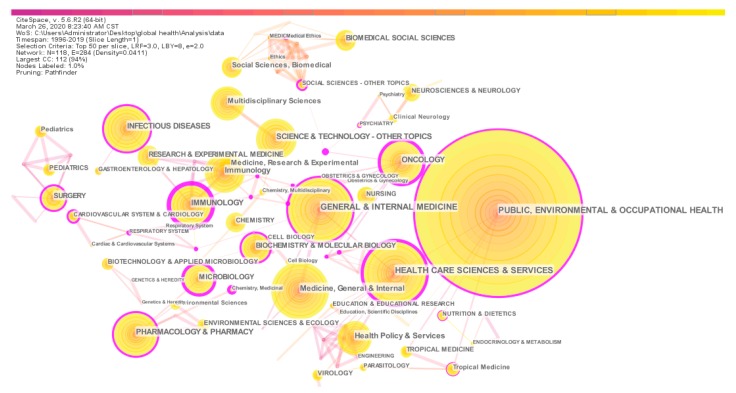
Knowledge map of co-occurrence categories.

**Figure 10:**
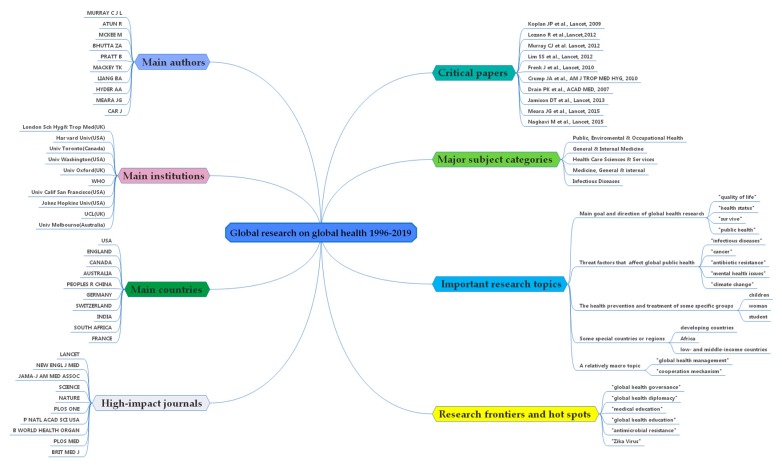
A comprehensive knowledge map in the global health research field: 1996–2019.

Co-occurrence analysis of subject categories allows us to intuitively understand the main subjects involved in a research field [48]. The classification of the categories in this study comes from the WOS database. In Figure 9, the purple circle on the edge of the node circle indicates that this node has a high intermediate centrality value. In Table 8, we list the top ten subject categories with co-occurrence frequency. Based on Figure 9 and Table 8, the subject categories of global health research are mainly “Public, Environmental and Occupational Health”, “General and Internal Medicine”, “Health Care Sciences and Services”, “Medicine, General and internal” and “Infectious Diseases”. It shows that global health research involves multiple disciplines and fields. Based on the analysis of the previous sections, we have drawn a comprehensive knowledge map of global health research, as shown in Figure 10. 

## 4. Discussion

### 4.1. General Information

In the analysis of scientific output, we find that the literature on global health research shows a trend of increasing nominally year by year. At the same time, the increase in the number of proceedings papers reflects, to a certain extent, the increase in international academic conferences in the global health field. It shows that international experts and scholars have paid continuous attention to the field of global health. In recent years, there were constant outbreaks of infectious diseases (Ebola Hemorrhagic Fever, MERS, Zika, etc.), increased mortality from chronic diseases, ethnic conflicts, and poverty. All of these have aroused widespread international concern and thinking about global health.

Among the five high-impact journals in the global health field, LANCET, NEW ENGL J MED, and JAMA-J AM MED ASSOC are three internationally recognized top journals in the medical field. These three journals have published for more than a century, and they have played a significant role in the history of human medicine. SCIENCE and NATURE are international comprehensive science magazines with a high reputation in academia. Most of the early basic literature and research hotspots in global health research come from these high-impact journals. Scholars in the global health field should pay attention to the scientific achievements published by these journals in real-time. 

This article explores scientific research cooperation in the global health field from three perspectives: Micro-author cooperation network, meso-institutional cooperation network, and macro-national cooperation network. Although there are certain academic exchanges and cooperation between authors, institutions, and countries in the global health field, these scientific research collaborations mostly occur between different institutions in a certain country and between significant scholars in an institution. In this field, developed countries (USA, England, Canada, Australia, Germany, etc.) still hold the leading position, while some developing countries (China, India) with a relatively large number of articles have not yet appeared prominent research institutions and scholars. Therefore, more scientific research exchanges with developing countries will be more conducive to the development of global health compared with developed countries taking measures such as medical aid and financial contributions to some developing countries with severe public health problems.

### 4.2. Research Topics and Emerging Trends

This study explores the research topics and emerging trends in the global health area mainly from two aspects: Keyword co-occurrence analysis and literature co-citation analysis. Keywords can clearly and intuitively reflect the research theme, core research content, and main research direction of an article. Therefore, in the fields of scientific text mining and scientometrics, the co-occurrence analysis of keywords can quickly grasp the development trends and research topics of a specific research field [49]. From the results of keyword co-occurrence analysis, there are five main research topics in the global health field, including global health goals and directions, research on global health risk factors, research on specific groups and specific countries or regions, and research on cooperative communication mechanisms. The goal of global health is to improve the equity of global health and the quality of life of people worldwide [50]. The original intention of the rise of global health is based on its effective way of dealing with global health inequity, which provides a new perspective and approach for achieving the goal of global health equity [51]. Global health focuses on the fairness and health influencing factors of global health, not just the health status and influencing factors of people in a specific country or region. Global health also pays attention to the global distribution of health and disease and its determinants, attaches importance to the impact of globalization on health and changes in the nature of global health governance, and emphasizes interdependence and coping strategies that transcend national and policy sector boundaries [52,53].

After literature citation analysis and cluster analysis, we learned that “global health governance”, “global health diplomacy”, “medical education”, “global health education”, “antimicrobial resistance” and “Zika Virus” are the current research frontiers and hot spots. Global health governance is a tool for dealing with the determinants of a healthy society across sectors [54]. The theoretical research and practical aspects of global health reflect that poor governance at the national and international levels will undermine the achievement of global health goals [55]. The catalyst for global health implementation is global health diplomacy. An important change in the 2000 G-8 summit was the linking of foreign policy with global health issues, and global health began to become a major goal and strategy of foreign policy. Global health diplomacy promotes the participation of various actors in the global governance actions taken to solve global health and related issues [56]. Some sovereign countries have established bilateral or multilateral global health strategies with other countries or organizations through mechanisms such as foreign policy and negotiation and consultation, formulated global health plans, and provided relevant financial and technical assistance to low- and middle-income countries [57].

Besides, “quality of life”, “energy balance; adipose tissue; appetite”, “subunit vaccine”, “mental illness”, “mental health”, “community impact”, “health services” and “interaction strategies” are important research topics and directions of global health. Combined with real-time news and past development experience, the current global outbreak of the new coronavirus in 2019 (COVID-19) has exposed many global health problems. Therefore, the prevention and control of infectious diseases, epidemiological studies of infectious diseases, international collaboration on global health, and health assistance will be a new research hotspot and emerging trends.

Despite the positive findings of the study, there are still some limitations. From the perspective of literature data, the literature data in this article only come from the Web of Science core collection database. Moreover, we only selected documents written in English. Secondly, this article does not contain grey literature, such as non-publicly published government documents, dissertations, non-publicly issued conference documents, scientific reports, technical archives, etc. From the perspective of visual analysis, this article does not interpret all the information in the knowledge graph. It is also one of the problems and directions that the follow-up research needs to think of and explore further.

## 5. Conclusions

In this study, based on the 14,692 literature data on global health research retrieved from the WOS core collection from 1996 to 2019, we conducted a scientometric analysis of the knowledge structure and knowledge field of global health research. At the same time, a visual analysis of the knowledge unit in the global health field was conducted, and a comprehensive knowledge map was drawn. Research in the global health field is extensive, involving multidisciplinary theories and methods, and its development requires the participation of researchers and new scholars in various fields. Scientific research cooperation between developed and developing countries in the global health field is particularly important and needs to be further strengthened. The mechanism of global health governance, the prevention and control of various infectious diseases, and the cultivation of professionals require long-term attention and discussion. This study provides researchers with an overview of global health through a systematic and comprehensive analysis of scientific output, core authors, significant institutions and countries, high impact journals, research cooperation networks, research topics, and emerging trends in the field of global health research. By presenting a new, comprehensive, and holistic knowledge map, this research contributes to the existing global health knowledge system. It also provides valuable guidance for researchers and related personnel to find the research direction and practice of global health.

## Figures and Tables

**Table 1:** The top 20 journals.

Rank	Journal	Cited Frequency	Impact Factor	Centrality	H-index
1	LANCET	7316	59.10	0.14	700
2	NEW ENGL J MED	4552	70.67	0.17	933
3	PLOS ONE	4401	2.77	0.00	268
4	JAMA-J AM MED ASSOC	3121	51.27	0.09	622
5	SCIENCE	3039	41.03	0.00	1058
6	NATURE	2924	43.07	0.20	1096
7	P NATL ACAD SCI USA	2860	9.58	0.14	699
8	B WORLD HEALTH ORGAN	2445	6.81	0.09	148
9	PLOS MED	2393	11.04	0.02	184
10	BRIT MED J	1955	27.60	0.07	392
11	SOC SCI MED	1929	3.08	0.03	213
12	J INFECT DIS	1741	5.04	0.28	231
13	AM J PUBLIC HEALTH	1686	5.38	0.06	236
14	CLIN INFECT DIS	1536	9.05	0.00	303
15	J BIOL CHEM	1529	4.10	0.50	477
16	LANCET INFECT DIS	1432	27.51	0.00	201
17	BMC PUBLIC HEALTH	1263	2.56	0.00	117
18	NAT MED	1229	30.64	0.06	497
19	AM J TROP MED HYG	1224	2.31	0.05	135
20	ANN INTERN MED	992	19.31	0.00	359

**Table 2:** The Top 20 authors.

Code	Author	Quantity	H-index	Centrality	Degree
1	MURRAY C.J.L.	31	120	0.04	49
2	ATUN R.	28	46	0.01	7
3	MCKEE M.	25	94	0.04	29
4	BHUTTA Z.A.	22	18	0.03	18
5	PRATT B.	20	12	0.00	2
6	MACKEY T.K.	18	21	0.00	1
7	LIANG B.A.	17	19	0.00	1
8	HYDER A.A.	17	24	0.00	1
9	MEARA J.G.	17	36	0.00	10
10	CAR J.	15	46	0.00	7
11	LABONTE R.	15	27	0.00	3
12	ADELOYE D.	14	15	0.00	9
13	YAYA S.	13	14	0.00	1
14	LEE K.	13	11	0.00	3
15	CELLA D.	13	122	0.00	3
16	RUDAN I.	13	12	0.01	16
17	ZEILER F.A.	12	15	0.00	8
18	SINGER P.A.	12	58	0.00	3
19	KHANG Y.H.	11	27	0.00	41
20	CAMPBELL H.	11	104	0.00	8

**Table 3:** The top 20 institutions.

Rank	Institutions	Publications	Centrality	Degree	HalfLife
1	London Sch Hyg and Trop Med (UK)	433	0.02	2	10
2	Harvard Univ (USA)	409	0.16	4	11
3	Univ Toronto (Canada)	396	0.18	11	17
4	Univ Washington (USA)	385	0.00	1	12
5	Univ Oxford (UK)	327	0.07	4	16
6	WHO	322	0.05	2	15
7	Univ Calif San Francisco (USA)	305	0.10	11	15
8	Johns Hopkins Univ (USA)	254	0.10	5	12
9	UCL (UK)	242	0.02	2	8
10	Univ Melbourne (Australia)	218	0.02	2	14
11	Emory Univ (USA)	206	0.06	3	9
12	Univ Sydney (Australia)	193	0.40	7	11
13	Columbia Univ (USA)	193	0.36	8	13
14	Univ Cape Town (South Africa)	191	0.12	7	11
15	Harvard Med Sch (USA)	186	0.02	2	2
16	Johns Hopkins Bloomberg Sch Publ Hith (USA)	183	0.02	2	7
17	Ctr Dis Control and Prevent (USA)	180	0.00	1	15
18	Yale Univ (USA)	177	0.04	3	17
19	Univ Penn (USA)	167	0.06	3	12
20	Duke Univ (USA)	166	0.04	5	17

**Table 4:** The top 20 countries.

Rank	Country	Publications	Percent%	Centrality	Degree	Burst	HalfLife
1	USA	6561	44.7	0.07	6	6.64	19
2	ENGLAND	2365	16.1	0.04	3	-	20
3	CANADA	1504	10.2	0.00	1	-	18
4	AUSTRALIA	1108	7.5	0.00	1	-	18
5	PEOPLES R CHINA	1086	7.3	0.01	3	-	17
6	GERMANY	759	5.2	0.00	2	7.05	18
7	SWITZERLAND	752	5.1	0.00	1	6.32	17
8	INDIA	635	4.3	0.00	1	-	16
9	SOUTH AFRICA	571	3.8	0.00	1	-	16
10	FRANCE	549	3.7	0.16	5	-	20
11	NETHERLANDS	527	3.6	0.00	2	-	19
12	ITALY	520	3.5	0.00	2	-	20
13	SWEDEN	425	2.8	0.02	4	7.61	19
14	BRAZIL	408	2.7	0.00	2	-	17
15	SPAIN	398	2.7	0.02	2	-	18
16	JAPAN	347	2.4	0.00	3	-	11
17	SCOTLAND	346	2.3	0.04	3	-	19
18	BELGIUM	333	2.3	0.17	7	-	19
19	SOUTH KOREA	308	2.1	0.00	2	-	16
20	KENYA	249	1.7	0.01	5	-	11

Note: Because of the scientific research cooperation between countries, two or more countries may appear in a paper. Therefore, when conducting a national cooperation network analysis, the total number of articles published in all countries is greater than 14692.

**Table 5:** List of keywords information.

Topic	Keyword	Frequency	Centrality	Degree	Burst
1	Global health	2583	0.26	13	-
	Quality of life	933	0.04	5	16.81
	Mortality	712	0.09	8	-
	Public health	535	0.12	7	-
	Survival	200	0.08	8	3.62
	Health status	52	0.20	6	13.38
2	Mental health	50	0.00	1	-
	Infection disease	779	0.11	10	-
	Hiv	368	0.11	8	-
	Cancer	355	0.07	8	-
	Obsity	348	0.02	3	-
	Tuberculosis	334	0.12	10	-
	Cardiovascular disease	149	0.05	3	14.94
	Mycobacterium tuberculosis	130	0.03	6	20.01
	Breast cancer	112	0.05	8	14.67
	Antibiotic resistance	39	0.00	1	-
	Human immunodeficiency virus	57	0.03	2	9.61
	Climate change	56	0.00	1	-
3	Children	518	0.00	1	-
	Woman	365	0.03	4	-
	Student	37	0.00	1	20.14
4	Developing country	399	0.06	6	11.81
	Afica	384	0.00	1	-
	United states	348	0.00	2	-
	Middle income country	62	0.00	1	-
5	Prevention	414	0.04	3	-
	Management	365	0.15	11	-
	Education	389	0.00	1	-
	Cooperation mechanism	244	0.00	1	-

**Table 6:** Top 10 cited documents.

Rank	Frequency	Author	Journal	Year	Burst
1	173	Koplan J.P. [2]	Lancet	2009	28.81
2	167	Lozano R. [39]	Lancet	2012	43.64
3	142	Murray C.J.L. [40]	Lancet	2012	38.32
4	130	Lim S.S. [41]	Lancet	2012	20.82
5	116	Frenk J. [42]	Lancet	2010	26.74
6	115	Crump J.A. [43]	AM J TROP MED HYG	2010	21.09
7	110	Drain P.K. [44]	ACAD MED	2007	29.31
8	105	Jamison D.T. [1]	Lancet	2013	22.87
9	92	Meara J.G. [45]	Lancet	2015	34.16
10	89	Naghavi M. [46]	Lancet	2015	25.82

**Table 7:** Summary table of cluster information.

Cluster ID	Size	Silhouette	Mean (Year)	Label (LLR)
0	162	0.719	2006	Global health governance; Global health diplomacy
1	127	0.827	2013	Status epilepticus; Antimicrobial resistance
2	66	0.889	2008	Medical education; Global health education
4	50	0.972	2001	Hepatitis c virus; Peptide inhibitors
5	47	0.999	1996	Quality of life;
7	44	0.981	2001	Susceptibility; Gene
8	41	0.943	2003	Energy balance; Adipose tissue; Appetite
9	40	0.963	2001	Subunit vaccine; Interferon-gamma
11	35	0.946	2005	Mycobacterium tuberculosis
13	23	0.967	2004	Mental illness; Mental health
14	20	0.991	2014	Zika Virus; Zika fever
15	20	0.997	2003	Bcg; Vaccine
17	18	0.98	2005	Mudpit proteomics; Egg secretome
21	14	0.994	2001	Community impact;
25	10	1	2005	Asthma; Rhintis
31	8	0.997	2000	Health services;
62	3	0.998	2007	General practitioner; Intervention strategies

Note: The silhouette value is the parameter used by CiteSpace software to evaluate the clustering effect. Specifically, the evaluation of clustering measuring the homogeneity of the network. The closer the silhouette value is to 1, the higher the homogeneity of the network and the clustering results with high reliability are greater than 0.7.

**Table 8:** The top 10 subject categories.

Rank	Category	Frequency	Centrality	Burst
1	Public, Environmental, and Occupational Health	2670	0.21	-
2	General and Internal Medicine	1155	0.29	-
3	Health Care Sciences and Services	1105	0.43	7.06
4	Medicine, General, and Internal	1047	0.01	3.33
5	Infectious Diseases	867	0.19	7.75
6	Pharmacology and Pharmacy	815	0.28	-
7	Immunology	783	0.73	-
8	Science and Technology-other topics	776	0.03	-
9	Oncology	773	0.47	3.35
10	Health Policy and Services	683	0.03	-
